# REFERRAL OF ASIANS TO SKILLED NURSING FACILITIES: FINDINGS FROM THE 2019 NATIONAL MEDICARE DATA

**DOI:** 10.1093/geroni/igad104.1739

**Published:** 2023-12-21

**Authors:** Hoang Nguyen, Chih-Ying Li, Huey-Ming Tzeng, Md Ibrahim Tahashilder, Yong-Fang Kuo

**Affiliations:** University of Texas Medical Branch at Galveston, Galveston, Texas, United States; University of Texas Medical Branch, Galveston, Texas, United States; University of Texas Medical Branch at Galveston, Galveston, Texas, United States; The University of Texas Medical Branch, Galveston, Texas, United States; University of Texas Medical Branch, Galveston, Texas, United States

## Abstract

A recent study of skilled nursing facilities (SNF) found Asian/Pacific Islanders are the most physically dependent and have the greatest cognitive impairment among all residents. Whether or not this is due to differential referral pattern to skilled nursing facilities is not known. We conducted a retrospective analysis of the 2019 national Medicare data to compare differences in referral to SNF among Asian, Black, Hispanic, and White adults ages 66 years and older following hospitalization for fracture (n=148,493), heart failure (n=377,398), joint replacement (n=509,347), pneumonia (n=258,614), urinary tract infection (n=195,816), or sepsis (n=518.619). Asians had the second lowest rates of SNF referral for all medical conditions examined, except for joint replacement. Hispanic had the lowest rates. For joint replacement, Asians had the highest rate of referral. The SNF referral rates among Asians compared to whites were 70.1% and 71.6% for fracture, 16.4% and 22.4% for heart failure, 16.8% and 21.2% for pneumonia, 30.1% and 34.6% for urinary tract infection (UTI), 29.3% and 32.5% for sepsis, and 32.1% and 23.4% for joint replacement. Asians had significantly lower odds of SNF referrals after hospitalization for fracture (OR=0.71; 95% CI: 0.65-0.77), heart failure (OR=0.51; 95% CI: 0.48-0.54), pneumonia (OR=0.46; 95% CI: 0.42-0.49), UTI (OR=0.59; 95% CI: 0.54-0.63), and sepsis (OR=0.55; 95% CI: 0.53-0.57), but significantly higher odds after joint replacement (OR=1.19; 95% CI: 1.12-1.26) compared to non-Hispanic White adults. The reason for the lower referral rates is unclear but could be from a lack of culturally appropriate facilities. More culturally appropriate facilities are needed.

